# Acupuncture as an Adjunct to Rehabilitation After Anterior Cruciate Ligament Injury and Reconstruction: An Anatomical and Neurophysiological Narrative Review

**DOI:** 10.7759/cureus.117053

**Published:** 2026-09-28

**Authors:** Dimitrios Kagkarakis, Alexandros Samolis, Dimosthenis Chrysikos, George Triantafyllou, George Tsakotos, Maria Piagkou

**Affiliations:** 1 Department of Anatomy, School of Medicine, Faculty of Health Sciences, National and Kapodistrian University of Athens, Athens, GRC

**Keywords:** acl reconstruction, acupuncture, anterior cruciate ligament, electroacupuncture, knee innervation, pain modulation, rehabilitation, surgical anatomy

## Abstract

Pain, effusion, quadriceps inhibition, and impaired proprioception are common challenges during recovery from anterior cruciate ligament (ACL) injury and reconstruction. While acupuncture is employed in certain rehabilitation protocols, evidence specific to ACL injury and ACL reconstruction (ACLR) remains limited and is frequently discussed alongside studies of chronic knee disorders. This narrative review investigates the surgical and neuroanatomical foundations of acupuncture around the knee, summarizes proposed neurophysiological mechanisms, and reviews the clinical literature most directly pertinent to ACL injury and ACLR. We conducted a comprehensive PubMed/MEDLINE search for studies involving acupuncture or electroacupuncture in conjunction with ACL injury or ACLR, supplemented by searches on knee sensory innervation, acupoint neuroanatomy, acupuncture safety, and higher-level evidence on knee pain. We also examined reference lists and cited articles. The extant literature on ACL/ACLR includes few small randomized or controlled trials, feasibility studies, a case report, and interventions using combined approaches. Some investigations report short-term improvements in pain, swelling, range of motion, or specific functional parameters; however, heterogeneity within the evidence base constrains definitive conclusions. Commonly utilized acupoints around the knee and leg are located proximate to periarticular tissues, muscle afferents, and identifiable peripheral nerves, rendering needle depth, direction, and regional anatomy clinically significant. Acupuncture may serve as an adjunctive modality for symptom management during ACL rehabilitation; however, current evidence does not support claims that it facilitates ligament repair, enhances graft incorporation, restores mechanical stability, prevents reinjury, or reduces the time required for a safe return to sport.

## Introduction and background

The anterior cruciate ligament (ACL) is a primary restraint to anterior tibial translation and contributes substantially to rotational stability of the knee. Its fiber bundles change tension with knee position, while mechanoreceptors and nociceptive free nerve endings contribute to proprioception and pain signaling [1]. Contemporary syntheses of knee sensory anatomy also show that the joint is supplied by a complex, overlapping network derived from femoral, sciatic, and obturator pathways, with considerable interindividual variability and a high density of nociceptors in the anterior capsule [2].

Rehabilitation remains central after ACL injury, whether management is non-operative or surgical. Following ACL reconstruction (ACLR), recovery requires progressive restoration of knee motion, quadriceps and hamstring function, neuromuscular control, proprioception, confidence, and sport-specific capacity. Recovery is multidimensional and commonly extends over many months rather than ending when pain or swelling subsides [3-15].

Researchers have studied acupuncture for musculoskeletal pain in perioperative or postoperative settings [16-26]. These findings require caution when applied to ACL rehabilitation. Evidence from knee osteoarthritis is not directly transferable to a traumatic ACL rupture or reconstructed graft, and a physiological response to needling does not necessarily translate into a clinically meaningful treatment effect. The present article is a narrative synthesis of anatomical, neurophysiological, and clinical evidence rather than an original anatomical investigation.

We therefore review the anatomy of the ACL and knee sensory network in relation to commonly used acupuncture points, outline traditional and biomedical approaches to acupuncture, summarize proposed analgesic mechanisms, and examine direct ACL/ACLR evidence separately from studies of other knee disorders.

## Review

Scope, search strategy, and study selection

This is a focused narrative review, not a systematic review or meta-analysis. We searched PubMed/MEDLINE from database inception through September 8, 2026. No lower publication-date limit was used. The main search combined Medical Subject Headings (MeSH) and title/abstract terms for acupuncture with terms for ACL injury and reconstruction. We applied no language or study-design filters during the initial search. The exact primary PubMed search strategy was: ("Acupuncture Therapy"(MeSH Terms) OR "Electroacupuncture"(MeSH Terms) OR acupuncture(Title/Abstract) OR electroacupuncture(Title/Abstract) OR "electro-acupuncture"(Title/Abstract) OR "acupuncture therapy"(Title/Abstract) OR "acupuncture treatment"(Title/Abstract)) AND ("Anterior Cruciate Ligament Injuries"(MeSH Terms) OR "Anterior Cruciate Ligament Reconstruction"(MeSH Terms) OR "Anterior Cruciate Ligament"(MeSH Terms) OR "anterior cruciate ligament"(Title/Abstract) OR "anterior cruciate ligament injury"(Title/Abstract) OR "anterior cruciate ligament injuries"(Title/Abstract) OR "anterior cruciate ligament reconstruction"(Title/Abstract) OR ACL(Title/Abstract) OR ACLR(Title/Abstract)). Additional PubMed searches covered knee and ACL sensory innervation, the anatomy and neuroanatomy of commonly used lower-limb acupoints, acupuncture-related adverse events, and recent systematic reviews, meta-analyses, and guidelines on knee osteoarthritis or chronic knee pain. We screened reference lists of key studies and reviews and used citation searching to identify relevant articles not captured by the main ACL search.

We prioritized human studies of ACL injury or ACLR; anatomical, cadaveric, histological, and neuroanatomical studies relevant to knee innervation or the structures underlying selected acupuncture points; and higher-level evidence to contextualize the limited ACL literature. The direct clinical synthesis excluded non-needle electrical stimulation, herbal treatment without an acupuncture component, animal studies, and papers outside the anatomical or rehabilitation scope. Because this is a narrative review, we did not apply PRISMA reporting, conduct a formal study-level risk-of-bias assessment, or perform quantitative pooling.

Surgical anatomy relevant to acupuncture around the knee

Anterior Cruciate Ligament (ACL) Anatomy, Innervation, and Proprioceptive Relevance

The ACL extends from the posteromedial aspect of the lateral femoral condyle to the anterior intercondylar region of the tibia. Its orientation and tension change with flexion and extension, allowing it to resist both translational and rotational loads. Neural elements within and around the ACL include mechanoreceptors and nociceptive free nerve endings. The wider knee sensory network includes branches from the saphenous and femoral systems, the tibial and common fibular divisions of the sciatic nerve, and obturator contributions [1,2,6,7].

Acupuncture around the anterior or lateral knee does not selectively reach the ACL. Depending on the point and depth of insertion, the tissues encountered include skin, subcutaneous tissue, fascia, muscle, periarticular connective tissue, and sensory nerve branches [2,6,8,9,12,13]. These anatomical relationships support somatosensory or neuromodulatory effects, but they do not provide evidence of direct mechanical or biological repair of a torn ACL or reconstructed graft [12-15,16-22].

Anatomical Basis of Commonly Used Knee and Lower-Limb Points

Standardized acupoint nomenclature improves reproducibility [5]. For clinical anatomy, however, the point name alone is insufficient; clinicians must also consider the underlying tissues and nearby nerves and vessels. Recent neuroanatomical studies have related stomach-channel points in the lower leg to branches of the deep fibular nerve and have shown that needle depth and direction can alter the structures encountered [8,9] (Table 1).

**Table 1:** Anatomical interpretation of commonly used knee and lower-limb acupuncture points. Standard point locations are based on established acupuncture nomenclature, while the anatomical relationships and safety considerations are derived from contemporary knee and acupoint neuroanatomy. The supporting references for each entry are provided in the final column. ACL: anterior cruciate ligament; ACLR: anterior cruciate ligament reconstruction.

Point	Standard topographic location	Relevant anatomical relationship	Surgical-anatomy comment	Supporting references
ST35 (Dubi)	Anterior knee, depression lateral to the patellar ligament with the knee flexed.	Patellar retinaculum, anterior capsule, and periarticular sensory tissues.	Periarticular point; do not describe it as direct ACL needling.	[2,5-7]
EX-LE4 (Neixiyan)	Anterior knee, depression medial to the patellar ligament.	Medial infrapatellar region, retinacular and capsular tissues; superficial sensory branches may be encountered.	Needling depth and postoperative wound location are important after ACLR.	[2,6,7,10]
GB34 (Yanglingquan)	Depression anterior and distal to the fibular head.	Close to the common fibular nerve and its branches.	This is a clinically important safety region because of the nearby common fibular nerve.	[5-7]
SP9 (Yinlingquan)	Medial proximal tibia below the medial tibial condyle.	Medial soft tissues near saphenous sensory pathways and proximal tibial structures.	Caution is appropriate with marked edema, altered sensation, or postoperative tenderness.	[5-7]
ST36 (Zusanli)	Anterolateral leg, approximately three B-cun inferior to ST35.	Dedicated neuroanatomical studies relate ST36 and neighboring stomach-channel points to the deep fibular nerve pathway; deeper levels approach the anterior tibial neurovascular compartment.	Needle depth and direction are important because deeper insertion approaches the anterior tibial neurovascular compartment.	[5,8,9]
ST34/SP10	Distal anterior and anteromedial thigh, respectively.	Quadriceps muscle and associated muscle afferents.	These points may be relevant to quadriceps-related pain or inhibition, although clinical benefit requires separate evidence.	[5,7,10]

Traditional Chinese and Western Medical Acupuncture

Traditional Chinese acupuncture is practiced within the diagnostic framework of Traditional Chinese Medicine, which uses concepts such as Qi, meridians, and individualized patterns of disharmony [10]. These concepts are part of the traditional explanatory system and are distinct from anatomical or physiological mechanisms.

Western medical acupuncture places needling within a biomedical model and relates its effects to peripheral sensory stimulation, spinal and supraspinal modulation, muscle and connective-tissue responses, and neurochemical pathways [11]. In rehabilitation, this approach allows treatment goals to be described using conventional outcomes such as pain, movement, muscle activation, and function.

In this review, traditional terminology is retained for point identification, whereas mechanistic and clinical claims are discussed according to the anatomical and clinical evidence available.

Plausible mechanisms of acupuncture-related analgesia

Peripheral and Spinal Mechanisms

Needling activates afferents in skin, muscle, and connective tissue. The pattern of sensory input depends on the site, depth, and stimulation method. A-delta and C-fiber activity may contribute to segmental spinal modulation and descending inhibitory control, and experimental studies also implicate endogenous opioid pathways [12,13]. The clinical contribution of these mechanisms varies with the condition being treated and the stimulation protocol.

Supraspinal Modulation

Functional imaging studies have described changes in brain networks involved in somatosensory, affective, and cognitive aspects of pain after acupuncture [14]. These observations are relevant to mechanisms, but they do not, by themselves, demonstrate therapeutic efficacy. Clinical benefit must be judged by patient-centered outcomes such as pain, range of motion, function, activity, quality of life, and return to sport.

Local Biochemical and Neuroimmune Effects

Needle manipulation can also produce local biochemical effects. In experimental models, adenosine A1 receptor signaling has been linked to local antinociception [15], while endogenous opioid pathways have been implicated in acupuncture-related analgesia [12,13]. These findings may help explain symptom modulation but do not show accelerated ligament healing, graft incorporation, or mechanical stabilization.

Clinical evidence in anterior cruciate ligament (ACL) injury and ACL reconstruction

*Direct Anterior Cruciate Ligament* *(ACL) and Anterior Cruciate Ligament Reconstruction ​​​​​​(*​*ACLR) Evidence*

The ACL-specific clinical literature is small and heterogeneous. Ding et al. randomized 140 patients after ACLR to routine rehabilitation with or without electroacupuncture. The electroacupuncture group had lower pain and swelling at one and three months and higher range-of-motion, International Knee Documentation Committee (IKDC), and Lysholm scores at several follow-up points [27]. Attrition, the single-country setting, and the specific seven-day treatment regimen limit how far these results can be generalized [16].

Barbosa et al. analyzed 22 patients who completed follow-up after ACLR and compared standard rehabilitation alone with rehabilitation plus nasal and cranial microsystem acupuncture. Pain at the graft-harvest site decreased in both groups, with a greater early reduction in the acupuncture group. The small sample size, the nonstandard microsystem approach, limited randomization, and the focus on donor-site thigh pain rather than the reconstructed ACL limit interpretation [17].

Cheng et al. randomized 40 patients undergoing ACLR to intraoperative electro-auricular acupuncture or no acupuncture during neuraxial anesthesia and sedation. The study primarily assessed whether blinding was feasible. Pain, nausea, opioid use, and satisfaction were similar between groups, although rescue nerve blocks were used less often in the acupuncture group. Because efficacy was not the primary aim, the study provides little information on longer-term rehabilitation outcomes [18].

Chang et al. reviewed 19 randomized trials involving 1,283 participants after ACLR. They reported possible improvements in pain, range of motion, swelling, and Lysholm score when Traditional Chinese Medicine interventions were added to conventional rehabilitation. The authors rated the evidence as low quality and noted substantial heterogeneity. Since several Traditional Chinese Medicine interventions were pooled, the reported effects cannot be assigned to acupuncture alone [19].

Deng et al. reported one patient with an acute ACL injury treated with deep electroacupuncture at BL39 and BL40. The case is of anatomical and procedural interest, but a single uncontrolled observation cannot establish efficacy or routine safety [20].

Xu et al. prospectively studied 90 patients with quadriceps atrophy after ACLR. Both groups received rehabilitation and growth hormone; the observation group also received electroacupuncture. The authors reported improvements in thigh atrophy index, pain, swelling, range of motion, Lysholm score, and stability measures at three and six months. The growth-hormone co-intervention limited methodological reporting, and the single-center design makes it difficult to determine electroacupuncture's independent effect [21].

Lu et al. conducted a randomized single-blind crossover trial in 20 men with previous ACL injury, comparing acupuncture at ST32, ST34, ST36, SP10, and BL57 with sham needling. Selected isokinetic measures improved immediately after real acupuncture, with reported effects lasting about 23-33 minutes. These short-term physiological findings in a small male-only sample do not establish a lasting rehabilitation or return-to-sport benefit [22] (Table 2).

**Table 2:** ACL/ACLR-specific acupuncture evidence. EA: electroacupuncture; ACL: anterior cruciate ligament; ACLR: anterior cruciate ligament reconstruction; ROM: range of motion; IKDC: International Knee Documentation Committee; TCM: traditional Chinese medicine.

Study	Design/sample	Clinical context	Intervention/comparator	Main finding	Key limitation
Ding et al., 2020 [16]	Randomized trial; 140 enrolled	Post-ACLR rehabilitation	EA + routine rehabilitation vs rehabilitation alone	Early pain/swelling benefit; higher ROM, IKDC, and Lysholm at several follow-ups	Attrition; intensive seven-day EA protocol; single-country setting
Barbosa et al., 2020 [17]	Controlled clinical trial; 22 completed	Early post-ACLR donor-site pain	Microsystem acupuncture + rehabilitation vs rehabilitation	Greater early thigh-pain reduction	Small sample; nonstandard microsystem approach; donor-site pain rather than ACL outcome
Cheng et al., 2021 [18]	Randomized feasibility trial; 40	Intraoperative ACLR	Electro-auricular acupuncture vs no acupuncture	Adequate blinding; no overall pain/opioid difference; fewer rescue blocks	Designed to test blinding feasibility rather than rehabilitation efficacy
Chang et al., 2021 [19]	Systematic review/meta-analysis; 19 RCTs, 1,283 participants	Post-ACLR rehabilitation	TCM interventions + conventional rehabilitation vs controls	Possible improvement in pain, ROM, swelling, and Lysholm score	Low-quality, heterogeneous evidence; several TCM interventions pooled
Deng et al., 2023 [20]	Case report; one patient	Acute ACL injury	Deep electroacupuncture at BL39/BL40	Clinical improvement described	Single uncontrolled case
Xu et al., 2024 [21]	Prospective comparative study; 90	Quadriceps atrophy after ACLR	EA + growth hormone + rehabilitation vs growth hormone + rehabilitation	Reported improvements in atrophy index and several knee outcomes	Growth-hormone co-intervention; limited methods reporting; single center
Lu et al., 2026 [22]	Randomized single-blinded crossover trial; 20 men	Previous ACL injury	Real acupuncture vs sham acupuncture	Immediate improvements in selected endurance/isokinetic outcomes	Small male-only sample; immediate rather than durable physiological outcomes

Indirect Evidence From Knee Osteoarthritis (KOA) and Chronic Knee Pain

Evidence for acupuncture is much broader in knee osteoarthritis (KOA) and chronic musculoskeletal pain than in ACL injury. Recent systematic reviews and meta-analyses report improvements in pain and physical function in some patients with KOA, although the estimated effect varies with the comparator, protocol, and study quality [23,24]. The American College of Rheumatology/Arthritis Foundation guideline gives acupuncture a conditional recommendation for knee osteoarthritis [25]. An individual-patient-data meta-analysis also found a modest benefit for chronic pain across several conditions, but these results do not establish effectiveness after ACL injury or reconstruction [26].

This broader literature supports a possible analgesic role but cannot answer ACL-specific questions about ligament healing, graft maturation, mechanical stability, reinjury, or return to pivoting sport. Those outcomes require trials designed specifically for ACL injury and ACLR.

Clinical Integration and Safety

If acupuncture is used after ACL injury or reconstruction, it should remain an adjunct to a structured rehabilitation program [3,4,16-22]. Restoring knee extension, controlling effusion, progressively strengthening the quadriceps and hamstrings, neuromuscular training, functional testing, and individualized return-to-sport criteria remain central to rehabilitation [3,4]. Current evidence suggests acupuncture is most reasonably used for short-term symptom control when pain or discomfort interferes with participation in rehabilitation [16-26].

Safe practice requires sterile technique, knowledge of local anatomy, appropriate needle depth and direction, and attention to the postoperative state [2,6-9,28]. Avoid needling through surgical wounds or suspected infection. Use extra caution with altered sensation, marked edema, increased bleeding risk, or anticoagulant therapy [28]. The region around the fibular head and the deeper anterolateral leg deserves particular attention because important nerves and vessels lie nearby [2,6-9].

In a systematic review and meta-analysis of prospective studies, serious acupuncture-related adverse events were uncommon, while minor events such as local bleeding and pain were more frequent [28]. Reporting methods varied considerably across the included studies. Thus, a generally favorable safety profile does not remove the need for appropriate training and anatomical knowledge.

Methodological limitations of the evidence

A strength of this review is its narrow focus on acupuncture in ACL injury and ACLR rehabilitation. The review presents direct ACL evidence separately from studies of KOA and chronic knee pain, and it does not present proposed mechanisms as proof of clinical efficacy.

The review also relates commonly used acupuncture points to the contemporary sensory anatomy of the knee and leg. This makes needle location, depth, direction, body habitus, and postoperative anatomy part of the clinical discussion rather than treating acupoints as isolated surface landmarks. It also identifies important outcomes with limited evidence, particularly ligament or graft healing, mechanical stability, reinjury, and return to sport.

ACL literature is also limited by small samples, heterogeneous study designs, the concentration of much postoperative evidence in a few settings, incomplete reporting of acupuncture technique and adverse events, and limited standardization of surgical and rehabilitation variables [16-22]. Evidence remains sparse for outcomes that matter most in ACL rehabilitation, including graft maturation, instrumented laxity, reinjury, validated return-to-sport readiness, and sustained sports participation [16-22].

This review has its own limitations. It is narrative rather than systematic, does not include formal risk-of-bias grading, and uses selected evidence from non-ACL knee conditions to provide mechanistic and clinical context. The conclusions should therefore be read as a qualitative synthesis rather than a pooled estimate of treatment effect.

Strengths of the present review

A strength of this review is its narrow focus on acupuncture in ACL injury and ACLR rehabilitation. The review presents direct ACL evidence separately from studies of KOA and chronic knee pain, and it does not present proposed mechanisms as proof of clinical efficacy.

## Conclusions

Acupuncture has anatomical and neurophysiological mechanisms that could influence pain and sensory input around the knee. In the limited ACL-specific literature, some studies report improvements in early postoperative symptoms or short-term functional measures when acupuncture is added to conventional rehabilitation. The studies are heterogeneous, however, and do not support acupuncture as a treatment for ACL healing or as a replacement for established rehabilitation. At present, acupuncture may be considered an optional adjunct for symptom control rather than a treatment intended to modify ligament or graft healing. Future studies should use clearly described, anatomically reproducible protocols; appropriate control groups; systematic adverse-event reporting; longer follow-up; and outcomes relevant to ACL recovery, including strength symmetry, validated patient-reported measures, objective stability, return-to-sport testing, reinjury, and sustained sports participation.
